# Is there an effect of gender on phenotypical expression of genetically mediated sinus node dysfunction?

**DOI:** 10.1093/europace/euag177

**Published:** 2026-07-09

**Authors:** Juliette Burbot, Leslie Placide, Julie Proukhnitzky, Estelle Gandjbakhch, Laura Delsarte, Mathieu Granier, François Massin, Adeline Goudal, Philippe Chevalier, Albano C Meli, Nicolas Molinari, Vincent Probst, Antoine Delinière, Jean-Luc Pasquié

**Affiliations:** Department of Cardiology, Electrophysiology and Cardiac Stimulation, Hôpital Arnaud de Villeneuve, Montpellier University Hospital, Montpellier 34090, France; PhyMedExp, Univ. Montpellier, Inserm, CNRS, Montpellier 34090, France; Department of Cardiology, Electrophysiology and Cardiac Stimulation, Hôpital Arnaud de Villeneuve, Montpellier University Hospital, Montpellier 34090, France; Cardiology Department, Hôpital la Pitié Salpétrière, Paris, France; Cardiology Department, Hôpital la Pitié Salpétrière, Paris, France; Department of Cardiology, Electrophysiology and Cardiac Stimulation, Hôpital Arnaud de Villeneuve, Montpellier University Hospital, Montpellier 34090, France; Department of Cardiology, Electrophysiology and Cardiac Stimulation, Hôpital Arnaud de Villeneuve, Montpellier University Hospital, Montpellier 34090, France; PhyMedExp, Univ. Montpellier, Inserm, CNRS, Montpellier 34090, France; Department of Cardiology, Electrophysiology and Cardiac Stimulation, Hôpital Arnaud de Villeneuve, Montpellier University Hospital, Montpellier 34090, France; Cardiology Department, University Hospital of Nantes, Nantes, France; Service de Rythmologie, Hospices Civils de Lyon, Lyon, France; PhyMedExp, Univ. Montpellier, Inserm, CNRS, Montpellier 34090, France; CHU de Montpellier, Department of Statistics, Montpellier University, Montpellier, France; Cardiology Department, University Hospital of Nantes, Nantes, France; Service de Rythmologie, Hospices Civils de Lyon, Lyon, France; Department of Cardiology, Electrophysiology and Cardiac Stimulation, Hôpital Arnaud de Villeneuve, Montpellier University Hospital, Montpellier 34090, France; PhyMedExp, Univ. Montpellier, Inserm, CNRS, Montpellier 34090, France

Sinus node dysfunction (SND) is a common bradyarrhythmia, predominantly degenerative and age-related. The automaticity of the sino-atrial node (SAN) depends on complex interactions of ionic currents.^1^ Recently, genetic mutations have been increasingly identified in the pathogenesis of SND, particularly among younger patients without structural heart disease. Pathogenic variants in ion channel genes, notably *SCN5A*, *HCN4*, and *CACNA1D*, are the most frequently reported.^2^ Sex is known to influence the phenotypic expression of other inherited arrhythmias. However, the impact of sex on the phenotype of genetic SND remains unclear. We therefore aimed to investigate sex-dependent differences in a cohort of patients with genetically confirmed SND.

We conducted a retrospective, multicentre, observational study across four French national referral centres for genetic arrhythmias (Lyon, Montpellier, Nantes, and Paris). We included 43 patients (22 men, 21 women) (*Table 1*) among 37 families, carrying pathogenic variants in *SCN5A* (*n* = 35, 81.4%) or HCN4 (*n* = 8, 18.6%), with a documented clinical diagnosis of SND, defined by sinus bradycardia (resting sinus rate ≤60 bpm in an appropriate clinical context), sino-atrial block, or sinus arrest documented on 12-lead electrocardiogram (ECG) or Holter monitoring. No patients were receiving beta-blockers or calcium channel blockers at the time of diagnosis. Patients with prior cardiac surgery were not included. Genetic testing was performed at the discretion of the treating physician, typically in the presence of features suggestive of inherited arrhythmia (young age, conduction abnormalities, family history, or suspicion of inherited arrhythmia syndromes), using targeted gene panels or exome sequencing. In several patients, genetic testing was prompted by associated phenotypes such as Brugada syndrome, ventricular arrhythmias, left ventricular non-compaction (LVNC), or a family history of sudden cardiac death. Clinical, electrocardiographic, and imaging data were analysed, with comparisons according to sex.

**Table 1:** Characteristics according to gender

Variables	Men (*n* = 21)	Women (*n* = 22)	*P*-value
Demographics			
Age at diagnosis (years), mean ± SD	34.62 (± 20.76)	29.50 (± 18.70)	0.423
Mutated gene (HCN4), *n* (%)	3 (14.29)	5 (22.73)	
Mutated gene (SCN5A), *n* (%)	18 (85.71)	17 (77.27)	
**Clinical history**			
Symptoms of SND, *n* (%)^a^	12 (57.14)	17 (77.28)	0.141
Family history of sudden cardiac death, *n* (%)	4 (19.05)	4 (18.18)	> 0.999
Atrial flutter, *n* (%)	10 (47.62)	6 (27.27)	0.168
Atrial fibrillation, *n* (%)	4 (19.05)	4 (18.18)	>0.999
History of stroke, *n* (%)	2 (9.52)	0 (0.00)	0.221
Vasovagal syncope, *n* (%)	2 (9.52)	4 (18.18)	0.665
**Device implantation**			
Pacemaker or ICD implanted, *n* (%)	15 (71.42)	14 (63.64)	0.426
— Pacemaker (PMK), *n* (%)	11 (73.34)	10 (71.43)	
— Defibrillator (ICD), *n* (%)	4 (26.67)	4 (28.57)	
Age at implantation (years), mean ± SD	34.62 (± 20.76)	29.50 (± 18.70)	0.423
Atrial pacing percentage (%), median (Q1;Q3)	99.00 (44.00 ; 100.00)	99.50 (76.25 ; 100.00)	0.622
Ventricular pacing percentage (%), median (Q1;Q3)	8.10 (1.00 ; 40.50)	1.20 (0.00 ; 25.00)	0.356
**ECG parameters**			
Heart rate (bpm), mean ± SD	59.00 (± 13.48)	53.90 (± 11.77)	0.204
PR interval (ms), median (Q1;Q3)	202.00 (168.00 ; 240.00)	192.00 (145.50 ; 237.50)	0.444
QRS duration (ms), mean ± SD	119.44 (± 26.11)	100.05 (± 18.10)	**0**.**017**
QTc interval (ms), median (Q1;Q3)	421.00 (362.00 ; 460.00)	410.00 (385.50 ; 447.50)	0.886
Brugada pattern on ECG, *n* (%)	3 (14.29)	6 (27.27)	0.457
**Echocardiography**			
LVEF (%), mean ± SD	58.56 (± 4.97)	61.14 (± 10.63)	0.075
Left atrial volume (mL/m^2^), mean ± SD	33.34 (± 9.80)	40.33 (± 20.22)	0.433
Left atrial area (cm^2^), mean ± SD	19.32 (± 3.74)	20.33 (± 9.48)	0.812
Right atrial area (cm^2^), mean ± SD	25.40 (± 7.08)	18,75 (± 0.50)	0.119
**Cardiac MRI**			
MRI abnormalities, *n* (%)	4 (19)	3 (13.4)	
Missing, *n* (%)	15 (71.43)	17 (77.27)	
**Others**			
Ongoing cardiac treatment, *n* (%) ^b^	4 (19.05)	6 (27.27)	
History of ventricular arrhythmias, *n* (%)	3 (14.29)	5 (22.73)	0.697

^a^Symptoms among: syncope, fainting, dyspnoea, asthenia

^b^Ongoing treatment among: antiarrhythmics or beta-blocker

Mean age at evaluation was 49 (±20) years, with diagnosis occurring at a mean age of 32 years, comparable between women and men (34.6 ± 20.8 vs. 29.5 ± 18.7, *P* = 0.423). Symptoms, including asthenia, dyspnoea, syncope, or presyncope, were more frequently reported in women than in men (81% vs. 60%, *P* = 0.141). Women trended towards lower resting heart rates (53.9 ± 12 bpm vs. 59.0 ± 13 bpm; *P* = 0.204). In contrast, men exhibited significantly longer QRS duration compared to women (119.4 ± 26.1 ms vs. 100.1 ± 18.1 ms; *P* = 0.017).

Regarding atrial arrhythmias, history of atrial flutter was reported in 47.6% of men vs. 27.3% of women (*P* = 0.168). Atrial fibrillation (AF) was documented exclusively in the *SCN5A* subgroup (22.9%) and was absent in the *HCN4* subgroup (0% vs. 8.0%). A substantial proportion of SCN5A patients (*n* = 9, 25%) also exhibited features consistent with Brugada syndrome, all spontaneously, and six (18.2%) experienced ventricular arrhythmias. Left ventricular non-compaction was strongly associated with the *HCN4* genotype, affecting 62.5% (5 of 8) of carriers. Two male patients, both *SCN5A* carriers, had a history of stroke. Left ventricular ejection fraction was preserved overall (59.8 ± 8.1%).

Cardiac devices were implanted in 69.0% of patients, predominantly permanent pacemakers (21 of 29, 72%), with no significant difference between men (75.0%) and women (63.6%) (*P* = 0.426). Eight SCN5A carriers received ICD based on individualized arrhythmic risk assessment, including ventricular arrhythmias and/or family history of sudden cardiac death. In the *HCN4* subgroup, only 25% (2 of 8) required device implantation, whereas 77% (27 of 35) of SCN5A patients were implanted. Among implanted patients, atrial pacing dependency was high in both genders (median 99%). Ventricular pacing burden trended higher in men (8.1% vs. 1.2%; *P* = 0.356). All patients experienced symptoms improvement following implantation.

Overall, no statistically significant sex-related differences were observed in the phenotypic expression of genetic SND. However, we observed distinct sex-specific trends. While the device implantation was comparable between genders, men exhibited more pronounced conduction disorders (significantly longer QRS duration), whereas women presented with greater symptom burden and with more pronounced impairment of sinus node automaticity (lower resting heart rates), particularly within the *HCN4* subgroup.

These findings should be interpreted in the context of a highly selected cohort from tertiary referral centres, which may have enriched for patients with more severe phenotypes or additional indications for genetic testing. Importantly, the indication for genetic testing may have differed between men and women, potentially contributing to the observed differences. In some patients, genetic testing was motivated by additional phenotypic features such as Brugada syndrome, ventricular arrhythmias, or LVNC, and SND may not have been the sole determinant of testing. This should be considered when interpreting the clinical applicability of our findings.

In non-genetic bradyarrhythmias, SND is generally reported to occur more frequently in women.^3^ However, in genetic cohorts, male predominance has been described. Abe et al. reported that *SCN5A*-related SND affected predominantly males (approx. 80%) with an earlier onset.^4,5^ Conversely, Kyndt *et al.*^6^ and Aizawa *et al.*^7^ observed a female predominance for SND phenotypes in families carrying *SCN5A* mutations, noting that males in the same families were more likely to develop Brugada syndrome.

Sinus node dysfunction is frequently viewed as a degenerative disease of the elderly (occurring above 65 y.o.).^8^ In sharp contrast, the median age at diagnosis in our cohort was 32 years. In routine practice, sinus bradycardia is common, particularly in young or physically active individuals, and does not systematically warrant genetic evaluation. Our findings do not support systematic genetic testing in all patients with sinus bradycardia. However, genetic testing may be considered in younger patients with SND in the presence of additional features such as bradycardia related symptoms, conduction abnormalities, family history, or overlapping phenotypes including Brugada syndrome or other inherited arrhythmia syndromes.^9^ This supports the concept that SND may represent one manifestation within a broader SCN5A-related channelopathy spectrum. Our data support the 2021 expert consensus recommendations to consider genetic testing in patients with documented family history.^10^

In conclusion, while no significant sex-related differences were identified, sex-specific trends in phenotypic expression were observed in this cohort of genetically confirmed SND. These findings support the concept of genetic SND as part of a broader arrhythmic phenotype and underscore the need for careful clinical evaluation when considering genetic testing. Larger prospective studies are warranted to further clarify these observations.

## Data Availability

The data underlying this article cannot be shared publicly due to patient privacy and ethical restrictions. De-identified data will be shared on reasonable request to the corresponding author, subject to approval by the participating institutions and applicable ethical and legal requirements.
